# The therapeutic potential of thalassotherapy for enhancing well-being and reducing pharmaceutical costs in individuals with disabilities

**DOI:** 10.3389/fpsyg.2025.1614410

**Published:** 2025-09-18

**Authors:** Emanuela Resta, Madia Lozupone, Preethymol Peter, Lucia Brunone, Silvio Tafuri, Gennaro Mariano Lenato, Aurora Bonvino, Paolo Taurisano

**Affiliations:** ^1^Department of Methods and Models for Economics, Territory and Finance, Sapienza University of Rome, Rome, Italy; ^2^Department of Translational Biomedicine and Neuroscience (DiBraiN), University of Bari Aldo Moro, Bari, Italy; ^3^Department of Innovation Engineering, University of Salento, Lecce, Italy; ^4^CRAP Incontri srl Residential Unit, Putignano, Italy; ^5^Public Health Unit, Interdisciplinary Department of Medicine, Bari Policlinico Hospital, University of Bari Aldo Moro, Bari, Italy; ^6^“Frugoni” Internal Medicine and Geriatric Unit, Interdisciplinary Department of Medicine, Bari Policlinico Hospital, University of Bari Aldo Moro, Bari, Italy

**Keywords:** thalassotherapy, cognitive disabilities, psychological well-being, non-pharmacological therapies, healthcare cost reduction

## Abstract

**Introduction:**

Thalassotherapy has shown promising effects on mental and physical well-being. However, its application among individuals with intellectual disabilities, psychiatric disorders, and cognitive impairments remains underexplored. This study evaluates the therapeutic potential of thalassotherapy in reducing pharmaceutical costs and improving well-being among individuals with intellectual disabilities, psychiatric disorders, and cognitive impairments.

**Materials and methods:**

This is an interventional cohort study that involved 144 participants aged 18 to 64 from two residential facilities in Apulia, Italy in 8 weeks. The intervention utilized seawater-based therapies at a certified thalassotherapy center, incorporating muscle awakening, music therapy, and group workshops. Participants underwent psychometric assessments using the VADO, Hamilton Anxiety Rating Scale (HAM-A), and Beck Depression Inventory (BDI) before and after the treatment.

**Results:**

Thalssotherpy indicated significant reductions in anxiety and depression scores. Specifically, repeated measures ANCOVA revealed a notable decrease in HAM-A (*p* = 0.012) and BDI (*p* < 0.001) scores, independent of age, gender, education, or diagnosis. These improvements suggest enhanced emotional regulation, increased self-esteem, and greater social participation.

**Discussion:**

The findings align with previous research on water-based therapies, highlighting thalassotherapy’s holistic benefits. Importantly, the study demonstrates the potential to reduce pharmaceutical dependence, thereby lowering healthcare costs and minimizing medication side effects. Despite its promising outcomes, the research acknowledges limitations, including a relatively small sample size and lack of long-term follow-up. Future studies with larger, diverse populations and controlled comparisons are necessary to validate the sustainability and broader applicability of these findings. Overall, thalassotherapy emerges as a cost-effective, non-pharmacological intervention that can complement traditional treatments, offering a valuable approach to enhancing the physical and psychological health of individuals with disabilities.

## 1 Introduction

The daily life of individuals with intellectual disability, psychotic disorders, or cognitive impairment presents significant challenges (Bell et al., 2015; John et al., 2022; Ramakrishnudu et al., 2021). Furthermore, these individuals often face difficulties in maintaining self-determination, forming appropriate social relationships, and sustaining healthy self-esteem (Shogren et al., 2015; Shogren et al., 2017; Niemiec et al., 2017; Wehmeyer et al., 2017). These complications, resulting in diminished self-esteem and an inaccurate self-image, lead to increased healthcare costs for these individuals in terms of medication adherence, hospital admissions, and additional treatment requirements (Pepper et al., 1981; Hecht and Wittchen, 1988; Olfson et al., 2000). The tendency toward social isolation may necessitate prolonged pharmacological management and require continuous appropriate supervision by parents, relatives, caregivers, or healthcare professionals to facilitate patients’ transition toward an active lifestyle through such medication therapy (Cummings and Miller, 2004; Duffy et al., 2005; Langeland et al., 2007). Several methods are framed to connect and help them, but a proper protocol needs to be improved. Therefore, novel treatments and laboratory primes must be determined to provide humanitarian outlets capable of aiding the lives of the families through which these aversions place them under hardship (Hugo, 1997; Browne, 1999).

A promising intervention that can address these challenges is the use of seawater and marine climate therapy, known as thalassotherapy. Thalassotherapy—a term derived from the Greek words *thalassa* (sea) and *therapeia* (treatment)—refers to a set of therapeutic practices that utilize the marine environment for health purposes. It remains a widely used empirical approach in the management of various chronic conditions, particularly dermatological, rheumatic, and respiratory disorders (Munteanu and Munteanu, 2019). In its broadest application, thalassotherapy includes not only bathing in seawater, but also a range of marine-based treatments, such as seaweed and sand baths, controlled sun exposure, inhalation of marine aerosols, and other structured interactions with the coastal environment. When applied under appropriate supervision, these natural elements are believed to support physical and psychological well-being, enhancing the body’s natural healing processes (Maraver et al., 2011). The sea has long been recognized as one of humanity’s primary sources of healing since ancient times (Lucchetta et al., 2007; Antonelli and Donelli, 2024). One possible therapeutic modality is thalassotherapy, which uses seawater and the marine climate to provide preventive care and recovery benefits. Humanity has considered the sea one of its primary natural healing sources for millennia (Antonelli and Donelli, 2024). This practice represents one of the few approaches to support people’s quality of life, even though a significant part of health still relies on adequate pharmacotherapy. However, reporting requirements for parents, guardians, and healthcare providers have become increasingly burdensome. Several methods have been developed recently to identify and support these patients, but even standardized protocols with global acceptance still need refinement. In particular, the tendency toward loneliness and emotional instability represents a challenge that periodic medication-based follow-ups cannot resolve (Langeland et al., 2007; Almeida et al., 2020). Thus, the search for new therapeutic options to provide relief from suffering of patients and family members, while they are trying to accommodate themselves in a complex reality is pointed. In this context, thalassotherapy can give a reason for optimism (Munteanu and Munteanu, 2019) through the use of seawater and marine climate to treat people with intellectual disabilities (ID), psychiatric pathologies, and cognitive disorders.

Moreover, thalassotherapy encompasses a wide range of applications across clinical, preventive, and rehabilitative domains, reflecting its multifaceted therapeutic potential. Clinically, thalassotherapy has been effectively utilized in the management of respiratory diseases, including chronic bronchitis and asthma, where inhalation of mineral-rich marine aerosols helps to clear airways, reduce inflammation, and improve pulmonary function (Moses, 2012). It has also demonstrated efficacy in treating dermatological conditions such as psoriasis, atopic dermatitis, and vitiligo, with benefits attributed to the combined effects of seawater minerals, controlled ultraviolet (UV) exposure, and biogenic compounds released by marine vegetation, which together promote skin healing and immune modulation (Antonelli and Donelli, 2024). Furthermore, musculoskeletal disorders like osteoarthritis, fibromyalgia, and rheumatoid arthritis benefit from thalassotherapy’s anti-inflammatory and analgesic properties, supported by the hydrostatic pressure and thermal effects of seawater that reduce joint loading and facilitate mobility (Maccarone et al., 2021).

Beyond clinical symptom management, thalassotherapy serves a critical preventive function by enhancing immune regulation. Regular exposure to the marine environment and its natural elements—such as minerals, sunlight, and marine aerosols—can bolster immune system function, modulate inflammatory responses, and potentially reduce the risk or severity of chronic inflammatory diseases (Maccarone et al., 2023). This immunomodulatory effect is particularly relevant in populations vulnerable to systemic inflammation or autoimmune disorders.

In addition to physical health benefits, thalassotherapy offers significant rehabilitative advantages, particularly for psychological well-being and social integration. Immersion in natural “blue spaces” has been linked to reductions in stress, anxiety, and depressive symptoms through mechanisms involving sensory stimulation, parasympathetic activation, and enhanced mood (Antonelli and Donelli, 2024; Barassi et al., 2020). The structured group activities often incorporated into thalassotherapy programs encourage social engagement, fostering a sense of community and belonging, which is especially valuable for individuals with mental health conditions or disabilities facing social isolation. This dual impact on mind and body underscores thalassotherapy’s role as a holistic intervention that complements traditional medical treatments and supports long-term health maintenance.

The present study evaluates therapeutic benefits in patients with disabilities, through targeting whole-body wellness upon thalassotherapy-focused approach. We hypothesize that, after 8 weeks of thalassotherapy intervention, patients will demonstrate significant improvements in terms of anxiety and depressive symptoms, as well as overall functioning. Consequently, it may be also surmised that additional, potential cost-savings associated with a reduction in pharmaceutical consumption could also be achieved. In exploring this alternative treatment model, we therefore support current research endeavours, in terms of working toward a more integrated collaborative care approach to help persons living with disabilities and their families meet and manage these daily challenges.

Previous research has explored the effects of thalassotherapy on acute stress levels, with Chennaoui et al. (2018) highlighting reductions in cortisol levels following water-based therapies, particularly in contexts of acute stress. Morer et al. (2020) demonstrated the benefits of thalassotherapy in post-stroke patients, emphasizing improvements in psychophysical health through structured rehabilitation interventions. Additionally, the review by Antonelli and Donelli (2024) emphasized the general psychological and physical health benefits of water-based therapies, focusing on their role in reducing stress and enhancing overall well-being. However, these studies often focused on specific populations or physiological markers, leaving a gap in understanding thalassotherapy’s impact on mental health conditions like anxiety and depression in individuals with intellectual disabilities, psychiatric disorders, and cognitive impairments.

This study aimed to fill this gap by evaluating the effects of thalassotherapy on anxiety and depression, as well as functional performance, in a cohort characterized by mental disabilities. Unlike previous work, our study focused on understanding how these treatments impact emotional well-being adopting a transdiagnostic approach.

## 2 Materials and methods

### 2.1 Participant population

The study population was recruited from two distinct residential facilities in Apulia, Italy, which specialize in providing care for individuals with intellectual disabilities, severe psychiatric disorders, and cognitive impairments. The cohort included both regular residents of the facilities, who benefit from services such as education, rehabilitation, assisted home living, supported accommodation, and work opportunities, as well as individuals receiving healthcare support through home visits organized by the same facilities.

Participation was targeted to adults aged 18 to 64 years. Individuals with severe, comorbid physical disabilities associated to the primary psychiatric condition were excluded from the study. All participants were informed about thalassotherapy opportunities. Informed consent was obtained either directly from.

All participants were informed about thalassotherapy opportunities. Informed consent was obtained either directly from the participants or, in cases where they were unable to provide consent, from their designated caregivers.

### 2.2 Setting and intervention

The study was conducted as an interventional cohort study, consisting of participation in an 8-week-long thalassotherapy treatment. The subjects were examined before and following the intervention using a battery of psychometric instruments (see Psychometric evaluation section).

The thalassotherapy intervention was performed at the “Cala Monaci,” an institutional bathing establishment in Monopoli belonging to the “Lilla Flag.” It has certified equipment and facilities for people with disabilities. The thalassotherapy program was implemented over a period of 8 weeks during summer time, with sessions conducted six days per week, averaging 8 h per day. Each day was structured into a series of therapeutic modules, combining physical, sensory, and social components in a stable and inclusive outdoor environment (see Table 1). A total of six therapeutic modules were implemented in the whole program, as detailed in Table 1. Each therapeutic module 1–6 had an average 60-min duration.

**Table 1:** The weekly schedule of activities included in the thalassotherapy program.

Daily schedule	
8.00–9.00 AM	Muscle Awakening

Morning session		
9.00–12.00 AM	Module 1	Team Beach Games and Water Games
	Module 2 (+Individual Physiotherapy)	Drawing and Handmade Craft Workshop
	Module 3	Interactive Discussion Group

Lunch break		
Afternoon session		
3.00–7.00 PM	Module 4	Tales listening and photography workshop
	Musculoskeletal recovery	
	Module 5	Beach music activity (Team Listening and Singing)
	Module 6	Beach dancing activity
	Rest and relaxation	

Therapeutic modules which were implemented in the whole program.

Each day was structured into a series of therapeutic modules, combining physical, sensory, and social components in a stable and inclusive outdoor environment. Each variable therapeutic module 1–6 had an average 60-min duration. *Only one day/week (on Tuesday), module 2 was replaced by basal individual physiotherapy.

Morning activities began with a “Muscle awakening” module, breathing exercises and muscle awakening by the sea (30 min), followed by a regenerating walk along the coast (30 min), thereby allowing participants to benefit from the inhalation of marine air rich in salt and minerals. This was followed by a 3-h group session integrating hydrogymnastics and music activity, aimed at improving joint mobility, muscle tone, and body awareness through rhythmic movement and auditory stimulation, through 3 pre-scheduled therapeutic modules. A relaxing break and lunch (3 h) completed the morning schedule.

The afternoon was focused on psycho-social stimulation and neuromotor recovery. Activities included psychosensory group interactions, such as board games, shared readings, and guided discussions, followed by mobilization exercises and professional massages to promote musculoskeletal recovery. The day concluded with a rest and recovery session, including relaxation on a deckchair with music, a regenerating hot shower, and individual evaluations (1 h).

The intervention took place in a barrier-free therapeutic coastal environment, specifically designed to ensure accessibility and safety for individuals with disabilities. Key features included non-slip walkways suitable for wheelchairs, accessible showers, toilets, and changing rooms, and beach wheelchairs (e.g., “Job” models) to facilitate sea access. The site also provided Braille signage, reserved parking, and trained multidisciplinary staff to support participants throughout the day. The natural setting—featuring walkable sandy terrain and characteristic Apulian coastal rock formations—offered rich multisensory stimulation, contributing to the overall therapeutic experience. Throughout the eight-week thalassotherapy program conducted on a fully accessible beach, relatives and caregivers played a pivotal role in supporting both the therapeutic process and the emotional well-being of participants. Their active and continuous involvement significantly contributed to the success of the intervention.

Caregivers ensured daily support and encouragement, facilitating participants’ active involvement in the various recreational, motor, and social activities. Their presence during aquatic sessions, beach games, and group interactions allowed for smoother transitions, increased confidence, and reduced anxiety among participants. In collaboration with healthcare professionals, educators, and volunteers, caregivers also provided valuable insights into the specific needs and preferences of each individual, enabling the personalization of interventions and promoting a more inclusive experience.

Aligned with the project’s goals, many caregivers took on the role of promoting autonomy, gently encouraging participants to engage in new activities while remaining available for respectful and non-intrusive assistance. Additionally, caregivers often acted as communication mediators, particularly for individuals with cognitive or language barriers, helping to interpret behaviors, express preferences, and facilitate mutual understanding between participants and staff.

Importantly, their presence offered emotional stability, fostering a secure, familiar, and empathetic environment. This emotional scaffolding not only enhanced participants’ comfort and engagement but also strengthened the overall therapeutic atmosphere. The integration of caregivers into the daily routine of the intervention thus served both practical and relational functions, emphasizing the importance of involving families and support networks in rehabilitation programs aimed at individuals with disabilities.

### 2.3 Psychometric evaluation

Comprehensive demographic data and extensive baseline clinical information and overall level of function were collected for all study participants.

All participants took a standardized test before and after the 8-week treatment:

The VADO tool (Morosini et al., 1998) is a continuous compliance instrument created distinctively for persons with psychological sickness. The assessment is usually based on functioning in the past 30 days and repeated every few weeks (e.g., monthly or quarterly) to track progress in achieving rehabilitation goals. An essential singularity of the VADO is that it identifies not only problems and limitations but also abilities, implying some potentials upon which a rehabilitation project can be based, aiming to develop as much as possible to recover the most disadvantaged functions.

The Hamilton Anxiety Rating Scale (Hamilton, 1959) is one of the earliest assessment tools developed to measure the severity of anxiety symptoms. It is still widely used in both clinical practice and research. The scale consists of 14 items, each defined by a series of symptoms, measuring both psychic and somatic anxiety. Although the HAM-A remains extensively used as an outcome measure in clinical studies, it has been criticized for its occasional inability to differentiate anxiolytic from antidepressant effects and somatic anxiety from secondary somatic effects. The HAM-A does not provide a set of standardized questions. Nevertheless, the reported levels of scale reliability appear to be acceptable.

The Beck Depression Inventory (Beck et al., 1961) is one of the most widely used assessment tools in clinical settings. It is a valuable instrument due to its straightforward application and ability to evaluate the severity of a patient’s depressive symptoms. The BDI is extensively utilized in clinical practice for its numerous advantages, including its high reliability. Additionally, it allows for the differentiation between severe depressive disorder and simple low mood. The scale can be applied to individuals aged 13 and above, and it consists of a self-report assessment where the patient independently completes a questionnaire of 21 items. The BDI presents a series of questions that describe the most common clinical symptoms observed in psychiatric patients with depression, and the individual must self-evaluate the extent to which they identify with the provided scenarios.

### 2.4 Data analysis

Based on our original study protocol, an *a priori* power analysis was conducted using G*Power. Assuming a medium effect size (Cohen’s *d* ≈ 0.3), an alpha level of 0.05, and 90% statistical power, the minimum required sample size was calculated to be 117 participants. Data analysis was conducted using the Jamovi software (www.jamovi.it). Descriptive statistics were employed to describe the demographic data and the baseline clinical/diagnostic information, using appropriate means analyses for continuous variables and frequency/percentages for categorical variables, as relevant.

A repeated-measures ANCOVA was performed on anxiety and depression scores, with age, gender, education level, and diagnosis included as non-interest covariates. The statistical significance threshold was set at 0.05.

Signed informed consent was obtained from each participant/legal guardian/next of kin. This study was conducted in accordance with the principles of the 1964 Declaration of Helsinki. The study was approved by the Local Institutional Review Board (Ethical Committee number: 1801/CEL). Approval Code: 576–2024 – Approval Date: 2024, July 30th.

## 3 Results

### 3.1 Cohort

The sample included 144 participants, with an average age of 49.7 years (SD = 12.80), ranging from 21 to 76 years. Gender distribution was balanced, with 52.8% female and 47.2% male (see Table 2). Regarding educational background, most participants had completed middle school (61 individuals, 41.7%), followed by those with elementary education (46 participants, 31.9%). A smaller proportion had completed high school (30 participants, 20.8%), and only 7 participants (4.9%) held a degree. In terms of diagnoses, schizophrenia was the most common condition, affecting 70 participants (48.6%), followed by depression in 24 participants (16.7%). Other diagnoses included bipolar disorder (16 participants, 11.1%), obsessive-compulsive disorder (14 participants, 9.7%), post-traumatic stress disorder (8 participants, 5.6%), behavioral disorders (10 participants, 6.9%), and antisocial personality disorder (2 participants, 1.4%).

**Table 2:** Sample characteristics, including age and psychometric scores before and after the intervention.

Measure	N	Mean	SD	Min	Max
Age (years)	144	49.7	12.80	21	76
VADO pre	144	40.8	6.31	28	50
VADO post	144	40.8	6.44	28	52
HAM-A pre	144	23.9	4.12	14	30
HAM-A post	144	20.1	4.76	9	29
BDI-II pre	144	32.4	6.79	14	45
BDI-II post	144	27.5	5.96	10	38

VADO, *Valutazione delle Abilità e Definizione degli Obiettivi*; HAM-A, hamilton anxiety rating scale; BDI, beck depression inventory.

### 3.2 Repeated measures ANCOVA on anxiety score

**Figure 1:**
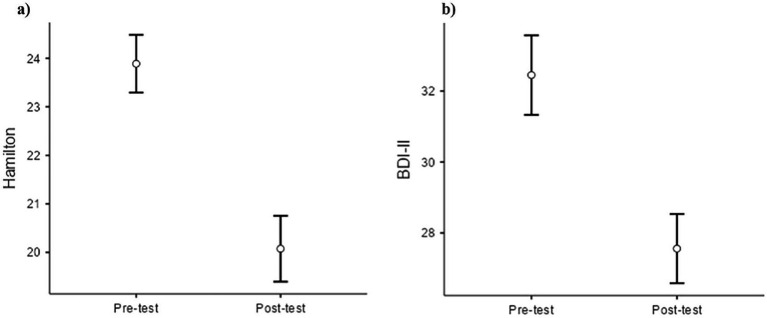
Repeated measures ANCOVA on Hamilton anxiety rating scale (HAM-A) **(a)** and beck depression inventory –II(BDI-II) Scores **(b)**, pre and post-treatment.

The analysis revealed a significant effect of time [*F* (1.139) = 6.41, *p* = 0.012], indicating reduced anxiety scores post-treatment. Between-subject effects demonstrated substantial contributions of age [*F* (1.139) = 29.65, *p* < 0.001] and education level [*F* (1.139) = 38.08, *p* < 0.001] to anxiety levels. No significant effects were observed for Gender or Diagnosis (Figure 1a).

### 3.3 Repeated measures ANCOVA on depression score

In the analysis of depression, we observed a highly significant main effect of time [*F* (1.139) = 23.44, *p* < 0.001], confirming a substantial decrease in depressive symptoms post-treatment. Between-subject effects showed no significant contributions of Age, Education, Gender, or Diagnosis to overall depression scores, indicating a consistent reduction in depressive symptoms across groups (Figure 1b).

### 3.4 Repeated measures ANCOVA on VADO score

The analysis of VADO scores did not reveal any significant differences between the pre-test and post-test scores, indicating that there were no statistically significant changes in VADO scores over time [*F* (1.139) = 0.41, *p* = 0.53].

The analysis was adjusted for covariates, including age, gender, education, and diagnosis. Results show a significant reduction in both anxiety and depression symptoms following treatment.

## 4 Discussion

According to the data collected during this study, thalassotherapy demonstrated promising effects on anxiety, depression, and functional performance in individuals with mental disabilities. These findings align with previous research on thalassotherapy, which has highlighted its benefits in reducing stress and enhancing psychophysical health in specific populations (Chennaoui et al., 2018; Morer et al., 2020; Barassi et al., 2020). However, our study extends this body of literature by adopting a transdiagnostic approach to evaluate its impact on emotional well-being across a broader cohort with diverse mental health conditions. The observed improvements in emotional well-being, coupled with enhancements in functional performance indicators such as balance and mobility, suggest that thalassotherapy could play a significant role in fostering greater independence and quality of life for individuals with intellectual disabilities, psychiatric disorders, and cognitive impairments. These results provide evidence for thalassotherapy as a potentially effective alternative or complementary intervention to traditional pharmaceutical treatments, especially in addressing anxiety and depression. By focusing on mental health outcomes and functional performance, this study fills a critical gap in the literature, offering a novel perspective on the role of water-based therapies in promoting emotional and physical well-being in populations often overlooked in previous research. These findings support the broader application of thalassotherapy as a holistic and accessible approach to improving mental health and overall quality of life.

According to Morer et al.’s research and other studies, these results agree with those obtained by other investigators who studied the effects of thalassotherapy in similar populations (Morer et al., 2020). Morer et al. focused on post-stroke patients, highlighting improvements in physical outcomes like balance and mobility through intensive thalassotherapy. In contrast, our study extends these findings by addressing mental health outcomes, such as anxiety and depression, in individuals with intellectual disabilities. While both studies underscore the benefits of thalassotherapy, our work adopts a broader transdiagnostic approach, emphasizing its potential for emotional well-being across diverse populations (Morer et al., 2020). Our study aimed to evaluate the effects of thalassotherapy on anxiety, depression, and functioning in individuals with mixed mental disorders. The results demonstrated significant reductions in anxiety and depression levels post-treatment. However, no significant changes were observed in functional score measured by the VADO scale, which remained stable throughout the study.

A significant reduction in anxiety was observed following the intervention, aligning with prior research that underscores the stress-reducing effects of water-based therapies. The sensory stimulation and relaxation provided by the aquatic environment, combined with structured group activities, likely contributed to this outcome. These mechanisms align with findings from Chennaoui et al. (2018), which demonstrated that thalassotherapy promotes parasympathetic activation and reduces cortisol levels, facilitating relaxation. The findings of this study also reveal that age and education level were significant contributors to anxiety levels, which highlights the importance of considering demographic factors in therapeutic interventions. This suggests that thalassotherapy might have varying effects depending on individual characteristics, but its general efficacy in reducing anxiety makes it a promising therapeutic option.

Thalassotherapy also led to significant reductions in depressive symptoms across all subgroups. Depression, often associated with feelings of helplessness and emotional distress, can be alleviated through interventions that promote relaxation, social connection, and sensory enrichment. The structured activities and immersive environment of thalassotherapy likely contributed to these improvements, creating opportunities for emotional relief and positive experiences. These results are consistent with findings by Barassi et al. (2020) and Antonelli and Donelli (2024), who reported that water-based therapies effectively improve mood and reduce depressive symptoms. Notably, the consistency of the depression score reduction across all subgroups—regardless of age, gender, education, or diagnosis—suggests that thalassotherapy has broad applicability and can be an effective intervention across diverse individuals.

These findings align with studies that emphasize the value of water-based therapies in improving psychological well-being (Barassi et al., 2020; Morer et al., 2020) and reinforce the idea that such therapies can serve as an adjunct to traditional pharmacological treatments, particularly in managing mental health conditions like anxiety and depression.

Several intertwined physiological and psychological mechanisms likely underpin the therapeutic effects observed in our population of individuals with disabilities. From a physiological perspective, immersion in mineral-rich seawater and exposure to coastal climate conditions have been associated with symptomatic improvements in dermatological (e.g., psoriasis, atopic dermatitis) and rheumatic disorders (e.g., fibromyalgia, ankylosing spondylitis), with notable reductions in disease severity and enhancements in quality of life (Antonelli and Donelli, 2024). These effects may be attributed to the combined action of mineral absorption, hydrostatic and thermal properties of seawater, and exposure to a mild, sunlit marine climate, which contribute to anti-inflammatory, circulatory, and muscle-relaxing benefits, as well as to the stimulation of vitamin D synthesis—a factor particularly relevant in populations with limited mobility. On a psychological level, the natural coastal environment offers unique therapeutic potential. For individuals with disabilities—who often face elevated risks of social isolation, stress, and reduced self-esteem—participation in structured, enjoyable, and inclusive outdoor activities supports emotional well-being, enhances social connectedness, and strengthens self-efficacy.

From an applied perspective, these results suggest that thalassotherapy could be an essential addition to the therapeutic repertoire for individuals with disabilities, particularly in the context of mental health management. By addressing both anxiety and depression, thalassotherapy offers a holistic treatment option that complements traditional pharmaceutical therapies. This is particularly valuable given the often complex medication regimens required for individuals with disabilities, which may include a range of psychotropic drugs that carry the risk of side effects. Thalassotherapy, being a non-invasive and natural intervention, provides an opportunity to reduce reliance on medications while still effectively addressing psychological symptoms. Moreover, the fact that thalassotherapy appears to reduce depression consistently across all considered demographic variables suggests that it could be integrated into treatment plans for a wide range of individuals with disabilities. Its broad applicability and ease of administration make it a feasible option for community-based and clinical settings. If thalassotherapy proves to be a sustainable intervention, it could be implemented more widely in rehabilitation programs, mental health clinics, and disability care facilities to improve the emotional well-being of individuals with disabilities, contributing to a more holistic approach to care (Antonelli and Donelli, 2024). Thalassotherapy may serve as a cost-effective, non-invasive intervention that enhances the quality and scope of traditional treatments for individuals with complex disabilities. The integration of thalassotherapy into rehabilitation settings may reduce pharmaceutical dependency, minimize side effects, and promote more holistic, person-centered approaches to care. Particularly, its potential to reduce pharmaceutical costs while improving quality of life makes it a valuable resource for both patients and healthcare systems. Such interventions may also alleviate caregiver burden by promoting patient autonomy and reducing the need for constant supervision, thereby generating positive ripple effects on family and healthcare systems.

However, while the findings are promising, several study limitations must be acknowledged. First, the relatively small sample size may limit the generalizability of the findings. Although the number of participants was sufficient to detect statistically significant effects, a larger cohort would enhance the reliability and external validity of the results, especially when analyzing subgroup differences based on diagnosis, age, or education. Second, the absence of a control group prevents us from definitively attributing the observed improvements to thalassotherapy alone. The lack of a randomized controlled design means that other factors—such as participant expectations, social interaction, or environmental novelty—may have influenced the outcomes. Third, the short duration of follow-up restricts our ability to assess the long-term sustainability of the observed benefits. It remains unclear whether the improvements in anxiety, depression, and functioning persist beyond the treatment period or whether repeated interventions are necessary to maintain gains over time.

## 5 Conclusion

The results represent an important step toward clarifying the benefits of thalassotherapy for people with mental disabilities. They suggest a viable therapeutic option in cases where pharmacological interventions might be limited or desired only to complement other strategies. The current study’s data indicate that thalassotherapy may improve psychological health outcomes, such as anxiety and depression. The results of this study indicate that thalassotherapy may be used as a cost-effective, non-pharmacological intervention to enhance the quality effect and general valuable characteristics regarding traditional treatment in people with mixed disabilities. These results serve as a basis for building more holistic treatment approaches integrating thalassotherapy to enhance this population’s quality of life and health outcomes. Future research should address these limitations by incorporating larger, more diverse samples and by using randomized controlled designs to isolate the specific effects of thalassotherapy. Longitudinal studies are also needed to evaluate the duration and durability of therapeutic outcomes. Furthermore, multicenter trials conducted in different geographical and clinical settings would enhance the applicability of the findings and support the development of standardized protocols for the use of thalassotherapy in populations with intellectual disabilities, psychiatric conditions, and cognitive impairments.

## Data Availability

The original contributions presented in the study are included in the article/supplementary material, further inquiries can be directed to the corresponding author/s.
